# Predictors of Online Patient Portal Use Among a Diverse Sample of Emerging Adults: Cross-sectional Survey

**DOI:** 10.2196/33356

**Published:** 2022-02-15

**Authors:** Julie A Wright, Julie E Volkman, Suzanne G Leveille, Daniel J Amante

**Affiliations:** 1 Exercise and Health Sciences University of Massachusetts Boston Boston, MA United States; 2 Department of Communication Bryant University Smithfield, RI United States; 3 Department of Population and Quantitative Health Sciences UMass Chan Medical School Worcester, MA United States; 4 College of Nursing and Health Sciences University of Massachusetts Boston Boston, MA United States; 5 Division of General Medicine Beth Israel Deaconess Medical Center Boston, MA United States

**Keywords:** internet, patient portal, emerging adults, portal, predictor, prediction, sample, cross-sectional, survey, usage, young adult, eHealth, literacy

## Abstract

**Background:**

Health self-management is increasingly being influenced by emerging health information technologies (IT), especially online patient portals. Patient portals provide patients with direct access to their health information, electronic tools to manage their health, and additional opportunities to engage with their care team. Previous studies have found that patient portal use is highest among patients with high eHealth literacy, the ability to find health information from electronic sources and apply the knowledge gained to solve a health problem. The role of eHealth literacy on patient portal use appears to be especially strong among older adults with chronic diseases. The use of patient portals among emerging adults (ages 18-29) is much less understood. Although generally healthy, emerging adults are more regular IT users and just beginning to independently navigate the health care system. A good understanding of how emerging adults are using online patient portals and what factors, including eHealth, impact portal use is lacking.

**Objective:**

The aim of this study is to describe patient portal use and explore the predictors of portal use among a diverse sample of emerging adults.

**Methods:**

A cross-sectional survey study that used convenience sampling was conducted at two universities. Data on demographics, health care encounters, eHealth literacy, patient engagement, and use of patient portal features (administrative and clinical) were obtained via self-report and summarized. Logistic regression models were used to examine factors associated with portal use.

**Results:**

Of the 340 emerging adults, 257 (76%) were female, 223 (65%) White, 156 (47%) low income, and 184 (54%) reported having patient portal access. Of those reporting access, 142 (77%) used at least 1 portal feature and 42 (23%) reported using none. Significant predictors were patient engagement (odds ratio [OR] 1.08, 95% CI 1.04-1.13, *P*=.001) and total encounters (OR 1.23, 95% CI 1.05-1.44, *P*=.009) but not eHealth literacy. Hispanic and Asian emerging adults were more likely to be frequent users of clinical portal features than White emerging adults (Hispanic: OR 2.97, 95% CI 1.03-8.52, *P*=.04; Asian: OR 4.28, 95% CI 1.08-16.89, *P*=.04).

**Conclusions:**

We found that about half of emerging adults had access to a patient portal. Among those with access, a majority reported using at least one portal feature. Factors associated with increased portal use included increased patient engagement and total clinical encounters. Self-reported eHealth literacy was not associated with patient portal use in this diverse sample of emerging adults. This may have been due to high overall eHealth literacy levels in this population of frequent IT users. There may also be racial/ethnic differences that are important to consider, as we found Hispanic and Asian emerging adults reported more frequent portal use than White emerging adults. Interventions to promote patient portal use among emerging adults should include strategies to increase awareness of portal access and engagement among patients with fewer clinical encounters, with a focus on preventative health management.

## Introduction

Patient self-management of their health is being promoted within health care organizations [1]. Health information technologies (IT) can provide opportunities to enhance patients’ self-management [2,3]. One IT that is increasingly adopted by health care systems is the online patient portal. The patient portal allows patients to access and manage their personal health information, request prescription refills, schedule appointments, and message with their health care team [4-6]. The increased availability of online patient portals provides patients with greater access to services and tools to engage in their health management [3,7-10]. Patient portals have demonstrated effectiveness, improving self-management of chronic conditions such as diabetes, hypertension, and depression [3,11] and increasing patient satisfaction [3,12]. Patient portal adoption and use has been shown to vary across patient populations, with increased use associated with patients of White race, those who speak English, and those who have private insurance [13,14]. Older patients and those living in rural areas with poor broadband access have also been shown to be less likely to use a patient portal [15]. An additional factor that could impact the use of patient portals is a patient’s eHealth literacy [16]. Extant literature suggests that as a patient’s eHealth literacy rises, their ability to use online health resources successfully also increases [3,17,18]. Interventions focused on improving patient portal use could include strategies to enhance eHealth literacy, especially among older patients who use technology less frequently and have greater health management demands due to chronic diseases. Whether such approaches would be helpful for younger, healthier patient populations is less clear.

This study is focused on emerging adults—specifically, higher education students aged 18-29 years old—and examines their use of secure online patient portals. Emerging adults represent a unique demographic, one in a state of transition from their dependency on parents/caregivers, yet not fully engaged in the responsibilities of adulthood [19]. For many emerging adults, university may represent their first independent experiences with the health care system [20]. They may have problems moving into the adult setting after parent-guided pediatric experiences; some may stop their health care altogether during this time of transition [20]. Furthermore, higher education students may have low eHealth literacy skills, despite their familiarity in using the internet to find health information [21,22].

Thus, it is important to understand emerging adults’ use of patient portals to inform tailoring of future interventions to improve their engagement with their care and health self-management. Understanding factors associated with online patient portal use will help guide development of interventions for this population. Enabling emerging adults to embrace new tools that increase access to health care services can expedite a culture change in which enhanced patient-provider partnerships lead to more effective care.

The purpose of this study is to describe the usage of online patient portal features among emerging adults. We hypothesized that eHealth literacy and patient engagement would be positively associated with reported portal use.

## Methods

### Recruitment and Ethics Approval

This cross-sectional survey study used nonprobability convenience sampling to recruit higher education students at a large, urban, public university with many minority students and a small, private university, both located in the Northeastern United States, to complete a cross-sectional survey during the months of March-May 2018 (Multimedia Appendix 1). Convenience sampling was conducted based on the norms associated with student recruitment at each university location and included students being invited via email to participate. For the public university setting, an email was sent via mass listserv to all undergraduate and graduate students. Students had the option to reply to the email and participate in the survey. For the private university setting, emails were sent to the faculty to forward to their students (undergraduate and/or graduate) to participate in the survey.

Email invitations contained a brief description of the purpose of the survey and an embedded survey link, using Qualtrics (April 2018) software, to complete on either a mobile device or desktop computer (web-based). Surveys were anonymous (no identifiers were linked to the respondents’ data), used no forced-choice questions, and offered voluntary entry into a random drawing for US $100. At the private university, faculty may offer extra credit as an incentive for participation; however, for this study, faculty members were instructed it was not needed due to the raffle incentive. All study procedures were approved by the UMass Boston and Bryant University Institutional Review Boards (2016148).

### Measures

#### Patient Portal Use

Patient portal use was assessed in two steps. First, participants were primed with an image of a typical health portal sign-in page and were asked to think about where they were currently getting their health care (Multimedia Appendix 2). Participants answered if their health care center offers a secure online patient portal (yes, no, and not sure). The second set of questions assessed their use of 8 common patient portal features. The stem was, “Have you ever used a patient portal for the following reasons?” followed by the 8 features, which were categorized as administrative or clinical in nature based on previous research [23]. The three administrative features included (1) check my appointment date or time, (2) make an appointment, and (3) request a prescription refill from my physician. The five clinical features were (1) check immunization records, (2) check lab results, (3) email provider, (4) read my visit notes (often called “doctor's notes”), and (5) post my own health information on the patient portal. Response options were (0) No, I don't have access, (1) No, I have never used, (2) Yes, I have used, and (3) Yes, I have used more than once. These items were developed specifically for this study and have not been validated against portal tracking information.

#### Health Care Utilization

Three items based on Lorig’s [24] healthcare utilization questionnaire were used to assess frequency of health care visits in the past 6 months. Participants were asked to report the frequency of visits to a physician’s office (not including hospitalizations), hospital emergency department, and urgent care center. The original items were modified by providing response options rather than using an open-ended format. Options ranged from 0 to 6 or more visits. Test-retest reliability has been reported for physician item (*r*=0.76) and emergency room item (*r*=0.94) [24,25].

#### eHealth Literacy

An 8-item modified version of the electronic Health Literacy Scale was used to assess participant eHealth literacy [26]. The modified version used items assessing the critical evaluative skills of information and frequency of engaging in these actions (behavioral literacy). The question stem was, “When looking for health information on the Internet, how often do you do the following?” A total of 8 behaviors were listed and participants were asked to respond on a 6-point frequency scale from never (1) to always (6). The eight behaviors were the following: (1) check the ownership of the health website, (2) check the website’s sponsor, (3) evaluate whether the health information is credible, (4) evaluate the credentials of the person providing the information on the website, (5) evaluate whether the coverage of the health topic is comprehensive, (6) check whether other print or web resources confirm the health information provided, (7) check whether the health information is up-to-date, and (8) discuss the health information with your health care provider.

#### Patient Engagement

The Altarum Consumer Engagement (ACE) Measure was used to assess patient engagement [27]. The ACE contains 12 items and 3 subscales with 4 items each: Commitment (eg, “I can stick with plans to exercise and eat a healthy diet”), Informed Choice (eg, “When choosing a new doctor, I look for official ratings based on patient health”), and Navigation (eg, “I have lots of experience using the healthcare system”). Responses were on a 5-point scale ranging from strongly disagree (0) to strongly agree (4) with a neutral midpoint. The scale has good construct validity with good internal reliability (Cronbach α) for the Informed Choice (α=.82) and Commitment (α=.85) subscales and fair reliability for Navigation (α=.66) [27].

#### Demographic Questions

Questions to characterize the respondents were asked at the end of the survey and included age, gender, race, ethnicity, language spoken at home, and years in the United States. Respondents were also asked to report on their parent or legal guardian’s annual income and the respondent’s employment and insurance status.

### Data Analysis Plan

Descriptive statistics were calculated to describe the sample’s characteristic and summary scores of variables of interest. The sample was defined by only those who self-reported 1 or more health care visits on the Lorig healthcare utilization questionnaire. The sample was further categorized by their use of the portal (nonuser versus user). Users were defined as those who reported using at least one of 8 portal features. Pearson chi-square analyses or independent *t* tests were used to examine differences by user status. A Levene test for equality of variances was run to determine the *t* statistic used to evaluate significance.

Multivariable logistic regression was used to examine correlates of portal use and to test our hypotheses that eHealth literacy and patient engagement would be positively associated with reported portal use. Covariates were entered into the model in five blocks: (1) demographics; (2) university type, insurance type, and health condition; (3) total encounters with health care system in last 6 months; (4) eHealth literacy score; and (5) patient engagement score. Participants who reported not having access to a health care portal or not using any patient portal feature were excluded from the analysis. Dichotomous variables were created for age (18-23 years old versus 24-29 years old), gender (female versus not female), ethnicity (Hispanic versus not Hispanic), university (public versus private), insurance (private versus not private), and health condition (condition versus no condition).

A second regression analysis was run to explore predictors of using portal features whose purpose is primarily clinical (eg, read visit notes) rather than administrative (eg, make an appointment) [23]. The outcome variable for this second analysis was dichotomized by high use (3 or more clinical features) versus low use (<3 clinical features). All analyses were completed in SPSS Statistics 25 (IBM Corp).

## Results

**Figure 1:**
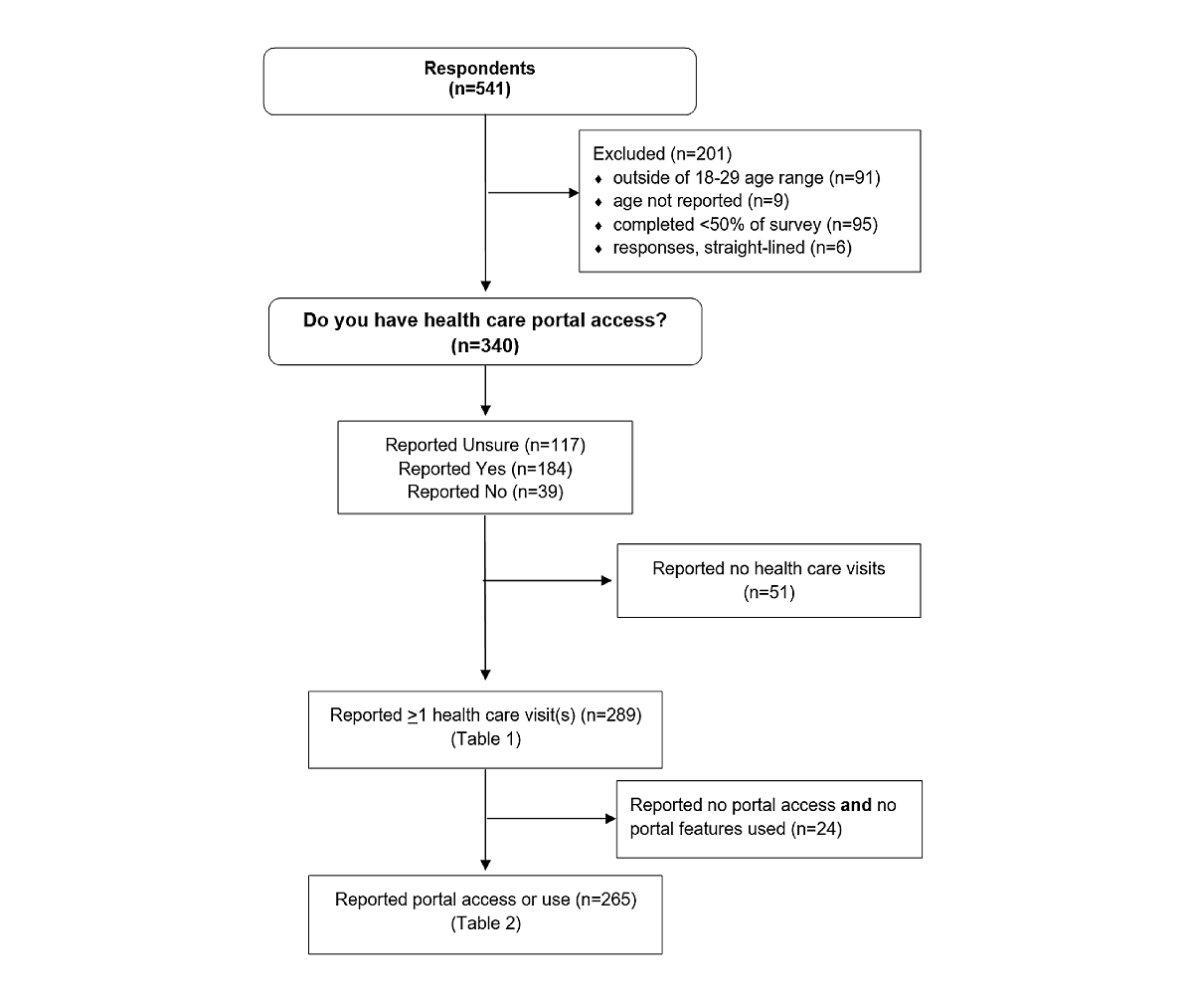
Sample flow chart.

Figure 1 displays a flow chart of how the analytic samples were defined. Email recruitment resulted in 541 students opening the link to the survey. After data cleaning and exclusions, the remaining analytic sample was 340 emerging adults (Figure 1). The majority of variables had 0-4 (1.2%) missing cases, with one variable missing 6 (2.1%). The majority of the 340 emerging adults (n=184, 54%) reported having access to a patient portal, while 11% (n=39) responded they did not and 34% (n=117) were unsure. We further reduced the sample to include only emerging adults who reported a health care visit in the previous 6 months (n=289). Table 1 describes this sample and classifies each participant as either a nonuser, defined as reporting using no features, or a user, defined as reporting the use of one or more portal features. Using this classification system, there were 124 nonusers and 165 users.

Of the 117 reporting they were unsure about having access to a patient portal, 70.1% (n=82) used none of the features while 29.9% (n=35) used 1 or more features—specifically, 5.1% (n=6) used at least 1 patient portal feature; 13.7% (n=16) used 2-3 patient portal features; and 11.1% (n=13) used 4 or more patient portal features. Of the 165 respondents who reported using portal features, the most used features were checking lab results (n=63, 38%), appointment time (n=61, 37%), and immunization records (n=58, 35%).

**Table 1:** Characteristics of students who reported a health care visit in the last 6 months and comparison of nonusers and users of the portal (N=289).

Variable		Nonuser^a^ (n=124)	User^b^ (n=165)	*P* value^c^	
**Age group (years), n (%)**					*.03*
	18-23	107 (86.3)	125 (75.8)		
	24-29	17 (13.7)	40 (24.2)		
**Gender, n (%)**					.30
	Female	92 (74.2)	131 (79.4)		
	Not female	32 (25.8)	34 (20.6)		
**Ethnicity, n (%)**					.46
	Not Hispanic	109 (87.9)	140 (84.8)		
	Hispanic	15 (12.1)	25 (15.2)		
**Race, n (%)**					.97
	White	82 (66.1)	111 (67.3)		
	Black	11 (8.9)	15 (9.1)		
	Asian	15 (12.1)	17 (10.3)		
	Other	16 (12.9)	22 (13.3)		
**Years residing in United States, n (%)**					.86
	0-5 years	9 (7.3)	14 (8.5)		
	More than 5 years	113 (91.1)	149 (90.3)		
	Prefer not to answer	2 (1.6)	2(1.2)		
**Parents' household income (US $), n (%)**					.95
	<40,000	36 (29.0)	45 (27.3)		
	41,000-80,000	39 (31.5)	54 (32.7)		
	>80,000	47 (37.9)	62 (37.6)		
	Missing	2 (1.6)	4 (2.4)		
**University type, n (%)**					*.002*
	Public	62 (50.0)	112 (67.9)		
	Private/not public	62 (50.0)	53 (32.1)		
**Insurance type, n (%)**					.38
	Private	109 (87.9)	139 (84.2)		
	Public	15 (12.1)	26 (15.8)		
**Do you have a health condition or disease that requires periodic visits or monitoring by a physician? n (%)**					.14
	No	98 (79.0)	118 (71.5)		
	Yes	26 (21.0)	47 (28.5)		
**Health care utilization in the previous 6 months,** **n (%)**					
	Physician visits (not including emergency department or urgent care)	1.73 (1.16)	2.11 (1.49)	*.02^d^*	
	Emergency department visits (nonuser n=123, user n=163)	0.22 (0.61)	0.36 (0.82)	.09^d^	
	Urgent care visits (nonuser n=121, user n=164)	0.48 (0.88)	0.59 (1.00)	.35	
	Total encounters (sum score)	2.41 (1.80)	3.05 (2.32)	*.009^d^*	
e-literacy (sum score, range 8-48), mean (SD)		26.33 (10.45)	27.15 (10.08)	.50	
Patient engagement (Altarum Consumer Engagement Measure sum score, range 12-60; nonuser n=123, user n=163), mean (SD)		37.89 (6.79)	41.83 (7.56)	*.001*	

^a^Nonusers were defined as those who reported not using any of the 8 portal features.

^b^Users were defined as those who reported using at least one of the 8 portal features.

^c^*P* values for Pearson chi-square test or *t* test. Italicized *P* values indicate significant difference between users and nonusers.

^d^Levene test *P*<.05, equal variances not assumed.

Multivariable logistic regression was used to examine the likelihood of being a user (n=165) versus nonuser (n=100) of the patient portal (Table 2). The respondents (n=265) included only those who reported a health care visit and reported access to a portal or reported they were unsure of access to a portal but reported use of at least one portal feature (Figure 1). Two cases from the sample of 265 were missing patient engagement scores (n=263). The final model included two significant variables, patient engagement (odds ratio [OR] 1.08, 95% CI 1.04-1.13, *P*=.001) and total encounters (OR 1.23, 95% CI 1.05-1.44, *P*=.009).

**Table 2:** Predictors of using portal features and predictors of high use versus low use of clinical features.

Variables in equation			Users vs nonusers^a^ (N=263)				High use vs low use of clinical features^b^ (N=263)		
			Adjusted odds ratio^c^		95% CI		Adjusted odds ratio		95% CI
**Gender**									
	Female	1.00				1.00			
	Not female	0.89		0.47-1.68		0.840		0.42-1.69	
**Age (years)**									
	18-23	1.00				1.00			
	24-29	1.43		0.67-3.03		0.63		0.31-1.28	
**Ethnicity**									
	Not Hispanic	1.00				1.00			
	Hispanic	1.13		0.40-3.20		*2.97*		*1.03-8.52*	
**Race**									
	White	1.00				1.00			
	Black	0.97		0.37-2.55		2.09		0.678-6.42	
	Asian	0.75		0.30-1.92		*4.28*		*1.08-16.89*	
	Other	0.61		0.21-1.79		2.52		0.630-10.05	
**University type**									
	Public	1.00				1.00			
	Not public	0.625		0.327-2.84		1.65		0.808-3.36	
**Health insurance type**									
	Private insurance	1.00				1.00			
	Not private	1.20		0.51-2.87		1.03		0.44-2.42	
**Health condition**									
	Yes	1.00				1.00			
	No	1.08		0.55-2.10		1.37		0.70-2.67	
Total health care encounters past 6 months			*1.23*		*1.05-1.44*		*1.16*		*1.01-1.34*
eHealth literacy score			0.99		0.96-1.02		0.97		0.94-1.00
Patient engagement score			*1.08*		*1.04-1.13*		*1.10*		*1.05-1.15*

^a^For this table, nonusers were defined as those who reported not using any of the 8 portal features and users were defined as those who reported using at least one of the 8 portal features.

^b^High users were defined as those who used 3 or more clinical portal features and low users were those who used less than 3 clinical portal features.

^c^Results from multivariable logistic regression models including all variables shown; significant relationships are italicized.

Using the same steps as the first logistic regression models, we examined the outcome variable of high versus low use of clinical portal features. Table 2 displays the results of the regression. Factors associated with high use of clinical portal features included Hispanic versus non-Hispanic ethnicity (OR 2.97, 95% CI 1.03-8.52, *P*=.04), Asian versus non-Asian (OR 4.28, 95% CI 1.08-16.8, *P*=.04), higher scores in patient engagement (OR 1.10, 95% CI 1.05-1.15, *P*=.001), and more health care encounters in the past 6 months (OR 1.16, 95% CI 1.01-1.34, *P*=.04).

## Discussion

### Principal Results

As hypothesized, patient engagement predicted online patient portal use, supporting previous research [7,9,28]. Findings also support that young adults have an interest in patient portals and engagement with their health care [29]. As they become more engaged, they are likely to find use of patient portals helps with medical decision-making [2].

We did not find that eHealth literacy was associated with patient portal use as we hypothesized. Others who found eHealth literacy was a correlate of patient portal use were reporting on samples with chronic conditions [3,30], compared to relatively healthy emerging adult populations. In our sample, participants had a midrange level of eHealth literacy (see Table 1). Our results echo previous studies’ findings that while emerging adults may be familiar with the internet and online information sources, they may not be engaging in health information seeking and thus have average eHealth literacy skills [22]. Interestingly, public university students had higher eHealth literacy scores than private university students, suggesting they can better evaluate eHealth resources than their counterparts. Public university students also had a higher number of health care visits compared to private university students, which may have contributed to greater familiarity with patient portals and influenced eHealth literacy. It will be important to investigate these relationships as emerging adults may engage in more health IT in coming years as portals become even more commonplace and their health management demands increase.

Analyses identified demographic and health factors associated with using clinical features of portals. The findings suggest that non-White emerging adults (specifically, Hispanic and Asian emerging adults), those having more health care encounters, and those with higher levels of patient engagement are using these features more frequently. A study of nearly 50,000 primary care patients showed that patients often look at clinical information through patient portals after a health care visit [23]. In addition, a recent report found patients from vulnerable populations, including those who were less educated, older, and from ethnic racial minority groups, were more likely to report benefits from reading clinic visit notes online compared to less vulnerable patients [31]. It is possible emerging adults from underserved communities may have more to gain from accessing clinical information from their online health records. Accessing patient portals may represent an important first step in taking control of one’s health, especially in the emerging adult population.

### Limitations

This study had a cross-sectional survey design and used convenience sampling. It is descriptive in nature and cannot determine causality regarding predictors of use. Although a nonprobability convenience sampling method reduces the generalizability of findings, it does allow researchers easier access to study participants and is a common practice in developmental and formative science [32,33]. Due to the nature of sampling, an exact response rate cannot be calculated. The sample size is also relatively small and further research is needed in larger higher education student populations across geographic regions. The study also did not assess if participants are sharing personal health information with others, such as parents or guardians. Furthermore, we did not collect information from faculty about whether extra credit was offered to students for participating, an approach that could bias the sample. Finally, the findings might not be generalizable to other emerging adult populations and health care settings.

### Conclusions

Online patient portals represent an important health IT tool offered by many health care organizations. In recent years, available features of patient portals continue to expand, giving patients greater access to their personal health information and tools to engage in self-management. This study adds valuable insight to continuing research into online patient portal use by bringing the emerging adult population into focus. There is still work needed to increase awareness of online patient portals among this age group, but there are also subpopulations that are already frequently using patient portals. Our findings suggest it is important to know more about the health IT available at public and private universities and that there is a need to further investigate eHealth literacy among this population. Interventions should focus on increasing patient portal awareness and engagement in health management in conjunction with improving eHealth literacy skills of the emerging adult patients.

## Appendix

Multimedia Appendix 1 Survey.

Multimedia Appendix 2 Example of patient portal.

## Abbreviations

ACE: Altarum Consumer Engagement
IT: information technology
OR: odds ratio
