# Supplementary material for: Predictors of Online Patient Portal Use Among a Diverse Sample of Emerging Adults: Cross-sectional Survey
Source: JMIR Form Res. 2022 Feb 15;6(2):e33356. doi: 10.2196/33356 (PMC8889472; doi:10.2196/33356)
Supplement: Multimedia Appendix 1 [file formative_v6i2e33356_app1.docx]

Project Seashore

Thank you for your interest in this survey! This research study is called, Advancing Student Healthcare. The survey will ask you about your opinions and past experiences with healthcare and using healthcare services, especially services available online.  The purpose of this research study is to gather information about university students’ attitudes and opinions to create a valid survey about eHealth and usage of healthcare services. Your participation may help us design surveys and programs that will help improve student health and healthcare.  Participating in this study *may* take up to 30 minutes of your time in total. After completing the survey, you will be entered in a random drawing for a $100 Amazon gift card.   There are no direct benefits from your participation.  Your decision whether to take part in this research study is voluntary.  You can stop at any time.  Your answers are confidential.  In fact, we will collect your name and contact information only if you would like to be entered into the gift card drawing.  Your name will not be linked to your answers. The information gathered for this research project will not be published or presented in a way that would allow anyone to identify you. Information gathered for this project will be stored in a secured, encrypted computer in the study office. Only the research team will have access to the data.  If you have questions, please contact Dr. X at phone number or email address. Please be aware that this survey may take 15 - 30 minutes to complete. You can stop and pick up where you left off if you need a break.

Age What is your age?

- Younger than 18 years old (1)
- 18-20 years (2)
- 21-23 years (3)
- 24-26 (4)
- 27-29 (5)
- Older than 29 years (6)

Skip To: End of Block If Age != 1 Screen Out:Thank you for your interest in Advancing Student Healthcare survey.  Unfortunately, you are not eligible to participate at this time

.

The first set of questions is about your experiences with healthcare. A reminder that all of your survey responses are anonymous and confidential.  For this survey, the words ‘physician or provider’ can mean either a nurse practitioner (NP), physician (MD), or physician's assistant (PA).

**In the past 6 months...**

|  | 0 visits (0) | 1 visit (1) | 2 visits (2) | 3 visits (3) | 4 visits (4) | 5 visits (5) | 6 or more visits (6) |
| --- | --- | --- | --- | --- | --- | --- | --- |
| How many times did you visit a physician? (Don't include visits while in the hospital or the hospital emergency room) (1) |  |  |  |  |  |  |  |
| How many times did you go to a hospital emergency room? (2) |  |  |  |  |  |  |  |
| How many times did you go to an urgent care facility? (Do not include emergency rooms) (3) |  |  |  |  |  |  |  |

Do you have a health condition or disease that requires periodic visits or monitoring by a physician?

- No (1)
- Yes, I have a disease or condition that requires periodic visits or monitoring. (Examples include but are not limited to diabetes, asthma, weight, anxiety, depression) (2)
- Prefer not to say (3)

Please read: Patient portals are secure, online websites that are hosted by a healthcare center and are linked to your medical records. Patients can: Access their medical information make appointments view lab results send e-mail messages to their healthcare providers access the notes written by a physician* ("doctor's notes") *not available on all portals

The picture below shows 1 example of a Patient Portal.  Other examples of patient portals include: Patient Site, MyHealthBeacon, MyChart, and MyLifeSpan Healthcare centers with portals include Partners, Beth Israel Deaconess, UHS, Boston Medical Center, Lifespan, Children’s Hospital, Tufts.


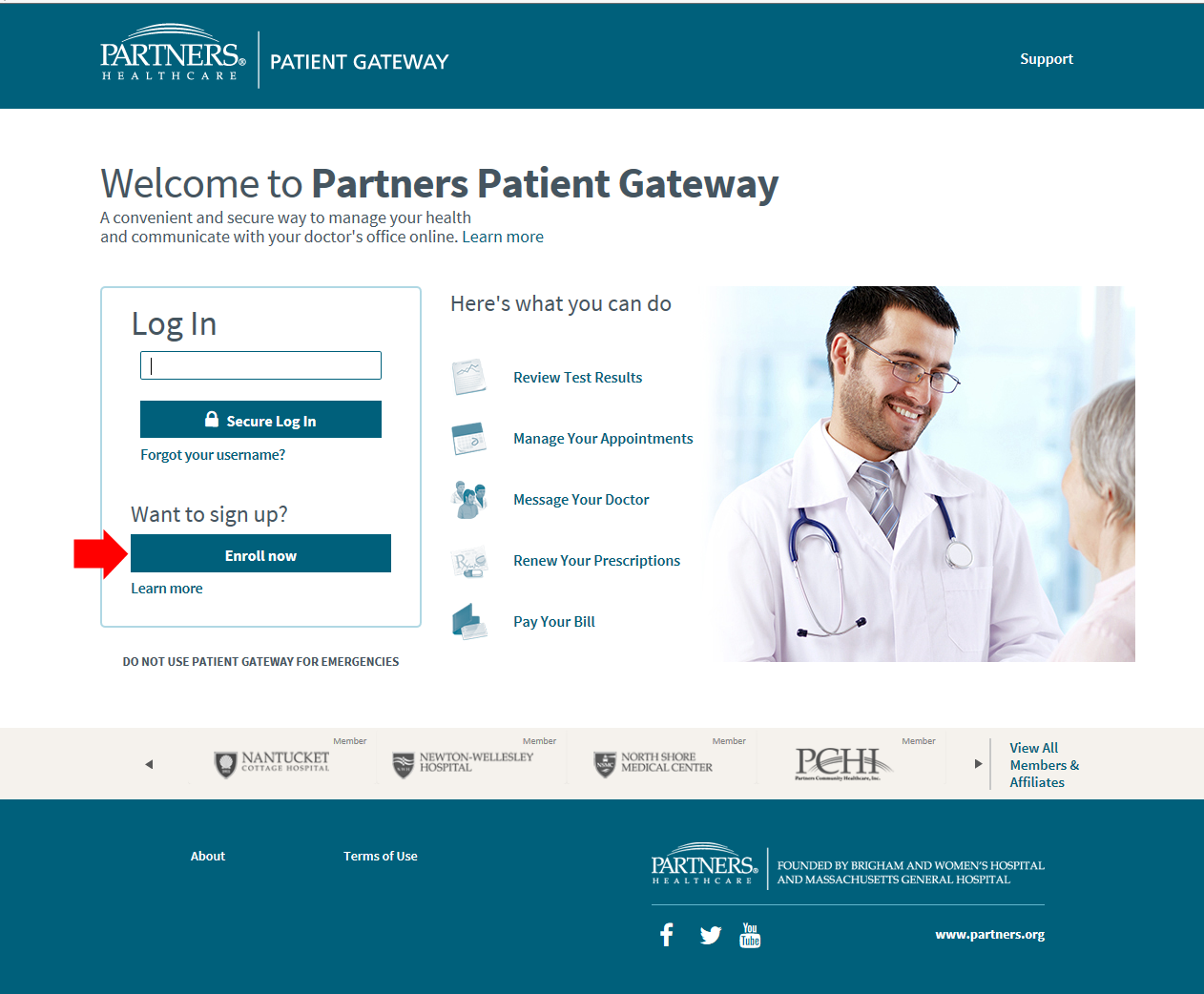


When answering the next set of questions, think about where you currently get your healthcare. 

Does your healthcare center offer a secure online patient portal?

- Yes (1)
- No (0)
- Not sure (3)

Have you ever used a patient portal for the following reasons?

|  | No, I don't have access (0) | No, I have never used (1) | Yes, I have used (2) | Yes, I have used more than once (3) |
| --- | --- | --- | --- | --- |
| Check immunization records (1) |  |  |  |  |
| Check lab results (2) |  |  |  |  |
| E-mail my healthcare provider (3) |  |  |  |  |
| Check my appointment date or time (4) |  |  |  |  |
| Make an appointment (5) |  |  |  |  |
| Request a prescription refill from my physician (6) |  |  |  |  |
| Read my visit notes, often called "doctor's notes" (7) |  |  |  |  |
| Post my own health information on the patient portal (8) |  |  |  |  |

**You are half-way done with the survey!**
The next 8 questions are about looking for health information on the Internet.  When looking for health information on the Internet, how often do you do the following...

|  | Never (1) | Almost never (2) | Sometimes (3) | Fairly often (4) | Very often (5) | Always (6) |
| --- | --- | --- | --- | --- | --- | --- |
| Check the ownership of the health website? (1) |  |  |  |  |  |  |
| Check the website's sponsor? (2) |  |  |  |  |  |  |
| Evaluate whether the health information is credible? (3) |  |  |  |  |  |  |
| Evaluate the credentials of the person providing the information on the website? (4) |  |  |  |  |  |  |
| Evaluate whether the coverage of the health topic is comprehensive? (5) |  |  |  |  |  |  |
| Check whether other print or Web resources confirm the health information provided? (6) |  |  |  |  |  |  |
| Check whether the health information is up-to-date? (7) |  |  |  |  |  |  |
| Discuss the health information with your healthcare provider? (8) |  |  |  |  |  |  |

The following 12 statements are about how you manage your health. Please rate how much you agree or disagree with the following statements

|  | Strongly disagree (1) | Disagree (2) | Neither agree nor disagree (3) | Agree (4) | Strongly agree (5) |
| --- | --- | --- | --- | --- | --- |
| I spend a lot of time learning about my health (1) |  |  |  |  |  |
| Even when life is stressful, I know I can continue to do the things that keep me healthy (2) |  |  |  |  |  |
| I feel comfortable talking to my doctor about my health (3) |  |  |  |  |  |
| When I work to improve my health, I succeed (4) |  |  |  |  |  |
| I have brought my own information about my health to show my doctor (5) |  |  |  |  |  |
| When choosing a new doctor, I look for information online (6) |  |  |  |  |  |
| I can stick with plans to exercise and eat a healthy diet (7) |  |  |  |  |  |
| I compare doctors using official ratings about how well their patients are doing (8) |  |  |  |  |  |
| I have lots of experience using the health care system (9) |  |  |  |  |  |
| When choosing a new doctor, I look for official ratings based patient health (10) |  |  |  |  |  |
| Different doctors give different advice; it's up to me to choose what's right for me (11) |  |  |  |  |  |
| I handle my health well (12) |  |  |  |  |  |

The last 10 questions are about you. A reminder that all of your survey responses are anonymous and confidential.

These questions will be used to report totals in a research report such as “50% of the survey respondents were female.” Your answers will never be used to identify you. In fact, we have no way of identifying who you are.

What is your gender?

- Male (1)
- Female (2)
- Transgender (3)
- Prefer not to answer (4)

What do you consider to be your racial background ? (You may select more than one choice.)

- African-American/Black (1)
- American Indian or Alaskan Native (2)
- Asian (3)
- Mixed Race (4)
- Native Hawaiian or Pacific Islander (5)
- White (6)
- Other (7)

Are you Hispanic or Latino or Spanish?

- No (1)
- Yes (2)
- Prefer not to answer (3)

What language do you speak at home primarily?

- Chinese (1)
- English (2)
- French Creole (3)
- Portuguese (4)
- Spanish (5)
- Vietnamese (6)
- Other (7) ________________________________________________
- Prefer not to answer (8)

How many years have you lived in the US?

- 0-5 years (1)
- 6-10 years (2)
- More than 10 years (3)
- Prefer not to answer (4)

Are you employed?

- Not employed (1)
- Part-time (2)
- Full-time (3)

What is your parent(s) or legal guardian(s) approximate household income?

- less than $20,000 per year (1)
- $21,000-40,000 per year (2)
- $41,000-60,000 per year (3)
- $61,000-80,000 per year (4)
- More than $80,000 per year (5)

Which university do you currently attend?

- Y Univerisity (1)
- X. University (2)
- Other (3) ________________________________________________

Which health insurance plan do you have?

- I am on my parent's health insurance (1)
- I have the university's health insurance plan (2)
- I have health insurance through employer or my spouse's employer (3)
- I have Mass Health (4)
- I do not know (5)

In the past 12 months, where did you go for your healthcare?  Check all that apply.

- Beth Israel Deaconess or Affiliated Community Health Centers (4)
- Boston Medical Center or Affiliated Community Health Centers (3)
- Lifespan or their Affiliated Community Health Centers (5)
- Partners Healthcare or Affiliated Health Centers (2)
- University Health Services (UHS) (1)
- Other (6) ________________________________________________

Thank you for participating in this survey about advancing student healthcare. We appreciate your time. 
Would you like to be entered into a random drawing?

- Yes (1)
- No (2)

To enter the random drawing, please click on the link below
