# Supplementary figures and images for: Predictors of Online Patient Portal Use Among a Diverse Sample of Emerging Adults: Cross-sectional Survey
Source: JMIR Form Res. 2022 Feb 15;6(2):e33356. doi: 10.2196/33356 (PMC8889472; doi:10.2196/33356)

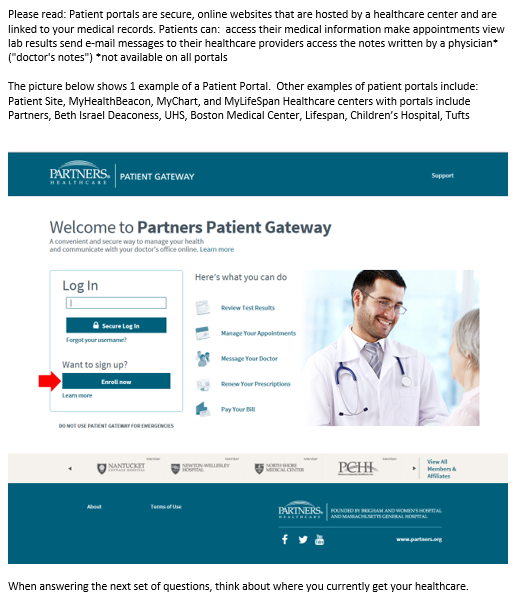

Supplement: Multimedia Appendix 2 [file formative_v6i2e33356_app2.png]
